# Increased Bone Marrow (BM) Plasma Level of Soluble CD30 and Correlations with BM Plasma Level of Interferon (IFN)-γ, CD4/CD8 T-Cell Ratio and Disease Severity in Aplastic Anemia

**DOI:** 10.1371/journal.pone.0110787

**Published:** 2014-11-10

**Authors:** Qingqing Wu, Jizhou Zhang, Jun Shi, Meili Ge, Xingxin Li, Yingqi Shao, Jianfeng Yao, Yizhou Zheng

**Affiliations:** Severe Aplastic Anemia Studying Program, State Key Laboratory of Experimental Hematology, Institute of Hematology & Blood Diseases Hospital, Chinese Academy of Medical Sciences & Peking Union Medical College, 288 Nanjing Road, Tianjin, 300020, P.R.CHINA; Beth Israel Deaconess Medical Center, Harvard Medical School, United States of America

## Abstract

Idiopathic aplastic anemia (AA) is an immune-mediated bone marrow failure syndrome. Immune abnormalities such as decreased lymphocyte counts, inverted CD4/CD8 T-cell ratio and increased IFN-γ-producing T cells have been found in AA. CD30, a surface protein belonging to the tumor necrosis factor receptor family and releasing from cell surface as a soluble form (sCD30) after activation, marks a subset of activated T cells secreting IFN-γ when exposed to allogeneic antigens. Our study found elevated BM plasma levels of sCD30 in patients with SAA, which were closely correlated with disease severity, including absolute lymphocyte count (ALC) and absolute netrophil count (ANC). We also noted that sCD30 levels were positively correlated with plasma IFN-γ levels and CD4/CD8 T-cell ratio in patients with SAA. In order to explain these phenomena, we stimulated T cells with alloantigen in vitro and found that CD30^+^ T cells were the major source of IFN-γ, and induced CD30^+^ T cells from patients with SAA produced significantly more IFN-γ than that from healthy individuals. In addition, increased proportion of CD8^+^ T cells in AA showed enhanced allogeneic response by the fact that they expressed more CD30 during allogeneic stimulation. sCD30 levels decreased in patients responded to immunosuppressive therapy. In conclusion, elevated BM plasma levels of sCD30 reflected the enhanced CD30^+^ T cell-mediated immune response in SAA. CD30 as a molecular marker that transiently expresses on IFN-γ-producing T cells, may participate in mediating bone marrow failure in AA, which also can facilitate our understanding of AA pathogenesis to identify new therapeutic targets.

## Introduction

Acquired aplastic anemia (AA) is an immune-mediated bone marrow (BM) failure syndrome characterized by persistent peripheral blood (PB) pancytopenia and BM hypoplasia [1]. Immune abnormalities such as decreased lymphocyte counts, inverted CD4/CD8 T-cell ratio and increased IFN-γ-producing T cells have been found in AA [2]–[4]. Autoreactive T cells activated by specific antigen(s) attacking CD34^+^ multipotential hematopoietic cells directly [5], and producing type I cytokines such as IFN-γ [6], are thought to be the major villain responsible for destruction of BM hematopoiesis in AA. Effectiveness of immunosuppressive agents further supports the immune-mediated pathogenesis of AA.

Although accumulating laboratory and clinical data suggest that AA is an immune-mediated disorder, the T cell-mediated immunopathology in AA remains to be poorly understood. Recent evidence indicates that oligoclonal expanded cytoxic T cells which are suggestive of an antigen-driven clonal response exist in AA [5], [7]. Furthermore, these oligoclones recognize and induce apoptosis of autologous myeloid cells [8]. However, the triggering autoantigens expressed by hematopoietic stem cells (HSC) in AA remain unknown. Only few reports identify autoantibodies in AA, and their pathological significance is unclear [9]–[12]. In a mouse model the single minor histocompatibility antigen H60 mismatch can trigger immune response and lead to massive BM destruction [13]. Other direct evidence to prove the existence of autoantigen in AA is still limited.

CD30, a cell-surface molecule belonging to the tumor necrosis factor receptor superfamily, is mainly expressed by activated T cells in the physiological condition [14]. CD30 is up-regulated on T cells exposed to allogeneic antigens, and these CD30^+^ T cells are a major source of IFN-γ [15]–[17]. Quickly after stimulation, surface CD30 is proteolytically cleaved by metalloproteinases and released into bloodstream as soluble CD30 (sCD30) [18]. Therefore, circulating sCD30 is thought to be reflective activation of the immune system.

Low serum levels of sCD30 are detected in healthy individuals [19]. In several classical autoimmune diseases, such as rheumatoid arthritis, atopic dermatitis and systemic lupus erythematosus, high levels of sCD30 have been found to represent the loss of tolerance to self-antigens [20]–[22]. More interestingly, sCD30 increases significantly in patients who developed acute graft versus host disease (GvHD) after allogeneic hematopoietic cell transplantation (HCT), which implies that elevated levels of sCD30 might be a potential biomarker of allograft rejection in HCT [23]–[24]. Brentuximab vedotin (SNG35), made by attaching the antitublin agent monomethyl auristatin E (MMAE) to the CD30-specific monoclonal antibody cAC10, has been proved to be efficient in inducing durable objective responses and resulting in tumor regression for CD30-positive lymphomas with only mild-to-moderate toxic effects [25]–[26]. The US Food and Drug Administration (FDA) has approved Brentuximab vedotin to be used in patients with relapsed/refractory Hodgkin lymphoma and anaplastic large cell lymphoma [27]–[28].

Thus, it's intriguing to probe whether CD30 is involved in over-production of IFN-γ by T cells given CD30^+^ T cells are the predominant proliferating and IFN-γ-producing cells in response to alloantigen. So, we carefully evaluated the role of CD30 in the pathogenesis of AA. Our findings suggested that CD30 as a cell surface marker that transiently expressed on activated T cells, might be associated with T cell-mediated bone marrow failure in AA, which could facilitate our understanding of AA pathogenesis to identify new therapeutic targets.

## Materials and Methods

### Patients and healthy individuals

We analysed samples of PB and BM from 56 patients with AA (median age 28 years, 29 male and 27 female), and 20 BM donors as healthy individuals (median age 27 years, 10 male and 10 female) after written informed consent in accordance with the Declaration of Helsinki, which was approved by the Ethics Committee of the Chinese Academy of Medical Sciences and Peking Union Medical College. The diagnosis and severity classifications of the cohort of patients with AA were established according to the international criteria [29]–[30], including 13 patients with non-severe AA (Non-SAA), 30 patients SAA and 13 patients very SAA (VSAA). Inherited bone marrow failures and clonal hematologic disorders were excluded from our study. Of 56 patients, 32 patients were analyzed at diagnosis, and 24 patients in complete response (CR) after immunosuppressive therapy (IST) of the combination of antithymocyte globulin (ATG) plus cyclosporine A (CSA). Serial samples pre- and post-ATG/CSA therapy were obtained from 6 patients with SAA. All patients were free from active infection at the time of sampling.

### Cell culture

BM mononuclear cells (BMMCs) isolated by Ficoll-Hypaque (1.077 g/mL) density gradient centrifugation were used for the measurements of CD30 mRNA expression. In some experiments, CD3^+^ T cells were enriched from BMMCs using a commercial CD3^+^ human T cell isolation kit (Mitenyi Biotec) according to the manufacturer's instructions. CD3^+^ T cells were mixed at a ratio of 1∶1 with autologous or allogenetic PB mononuclear cells pretreated with mitomycin C and seeded at 5×10^5^ cells/mL. Cells were cultured for 7 days at 37°C with 5% CO_2_ atmosphere, in an optimized serum-free cell culture medium (TexMACS Medium, Miltenyi Biotec) developed for the cultivation and expansion of human T cells, supplemented with 2 mmol/L penicillin/streptomycin. Every 24 h, cells and culture supernatants were collected and analysed by flow cytometry (FCM) and enzyme-linked immune sorbent assay (ELISA), respectively.

### Quantitative real-time PCR

Total RNA was isolated from cells with Trizol reagent (Invitrogen) following the manufacturer's instructions. cDNA was synthesized from the purified RNA using a reverse transcription system (Promega) with random primers. Real-time PCR was performed using SYBR green PCR kit (Invitrogen). β-actin was used to normalize gene expression levels. PCR primer sequences were as follows: CD30 forward 5′-GACAAGGCTGTCAGGAGGTG-3′, reverse 5′-ACTGGAGGTTGCTGGGGACA-3′; β-actin forward 5′- CTCTTCCAGCCTTCCTTCCT-3′, reverse 5′- AGCACTGTGTGTTGGCGTACAG-3′.

### Flow cytometry and cell sorting

In the total 7 days mixed lymphocyte culture system, surface antigen of T cells were analysed by FCM every 24 h. Cultured cells were harvested and washed twice in PBS by centrifugation at 300 g for 5 min. Cells were then stained with the following antibodies: CD3-FITC (Biolegend), CD8-APC (Biolegend), CD30-PE (eBioscience), and the appropriate isotypic control according to the manufacturer's instructions. After incubation, cells were resuspended with 1% paraformaldehyde for FCM analysis. Cell sorting was performed by using BD FACS Canto II (BD Biosciences).

### Cytokine ELISA

The plasma and culture supernatants were stored at −80°C until ELISA was performed. The concentrations of sCD30 and IFN-γ were measured by Human sCD30 Instant ELISA kit (eBioscience) and Human IFN-γ ELISA kit (Boshide Biotech, China), respectively.

### Statistics

Data were shown as mean ± SEM. The statistical differences were evaluated by the nonparametric Mann–Whitney U-test between unpaired data and by Wilcoxon matched pairs test for two paired variables. The Spearman's rank correlation test was used for correlation analysis. All analyses were performed using SPSS 16.0 software (SPSS Science). *P* values <0.05 were considered statistically significant.

### Ethics Statement

We analysed samples of PB and BM from 56 patients with AA, and 20 BM donors as healthy individuals after written informed consent in accordance with the Declaration of Helsinki to the protocol obtained, which was approved by the Ethics Committee of the Chinese Academy of Medical Sciences and Peking Union Medical College.

## Results

### Quantitative measurement of sCD30 in AA patients

**Figure 1:**
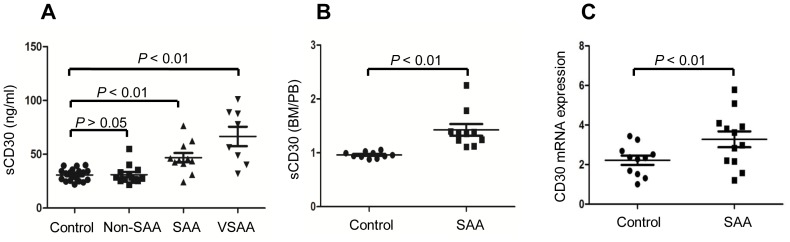
Plasma sCD30 levels and CD30 mRNA expressions in BMMCs from AA patients (n = 42) and healthy individuals (n = 20). (A) BM plasma sCD30 concentrations in healthy individuals (n = 20), Non-SAA patients (n = 13), SAA patients (n = 11) and VSAA patients (n = 8) were measured by ELSIA. (B) The ratios of BM plasma sCD30 level/PB plasma sCD30 level in SAA and VSAA patients (n = 11) and in healthy individuals (n = 11). (C) Relative expressions of CD30 in BMMNCs were measured by real-time PCR in *de novo* SAA patients (n = 12) and healthy individuals (n = 11).

The comparisons of BM plasma sCD30 levels between *de novo* AA patients (including 13 Non-SAA, 15 SAA and 14 VSAA) and healthy individuals measured by ELISA were shown in Figure 1A. Plasma sCD30 levels were found to be positively correlated with the severity of AA, the median plasma sCD30 levels in healthy individuals, Non-SAA, SAA and VSAA patients were as of 30 ng/mL, 32 ng/mL, 46 ng/mL and 62 ng/mL, respectively.

Because CD30 was suggested to highly express in the target organs of certain autoimmune diseases, we compared sCD30 levels between PB and BM (the latter as the target organ in AA) from 12 SAA and VSAA patients and 11 healthy individuals. Data shown that sCD30 level in BM plasma was higher than its corresponding PB level in each patient with SAA, but healthy individuals had comparable levels of sCD30 between BM and PB, as shown by the significantly higher ratio of BM sCD30/PB sCD30 in patients with SAA in comparison with healthy individuals (Figure 1B). To further confirm CD30 expression in BM, we analysed the mRNA levels of CD30 in BMMCs by real-time PCR, which revealed significantly increased CD30 expression in patients with SAA (Figure 1C).

### Correlations between BM plasma sCD30 levels and baseline PB counts

**Figure 2:**
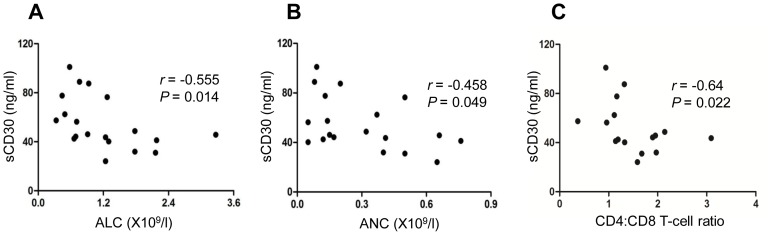
Correlations of BM plasma sCD30 levels with baseline ALC (A), ANC (B), or CD4/CD8 ratio (C) in AA patients (n = 19).

In consideration of alloactivated T cells are the major source of sCD30, we analysed the correlation between BM plasma sCD30 levels and baseline absolute lymphocyte count (ALC), absolute netrophil count (ANC) and absolute reticulocyte count (ARC) in patients with SAA (including VSAA). Our results showed specific inverse correlations between sCD30 levels and ALC (Figure 2A) or ANC (Figure 2B), although no correlation were found between sCD30 levels and ARC (Figure S4).

Interestingly, we also found an inverse correlation between sCD30 levels and CD4/CD8 T-cell ratio (Figure 2C), which suggested patient with a higher CD8^+^ T cell proportion tended to have a higher plasma level of sCD30.

### Correlation between BM plasma sCD30 levels and BM plasma IFN-γ levels

**Figure 3:**
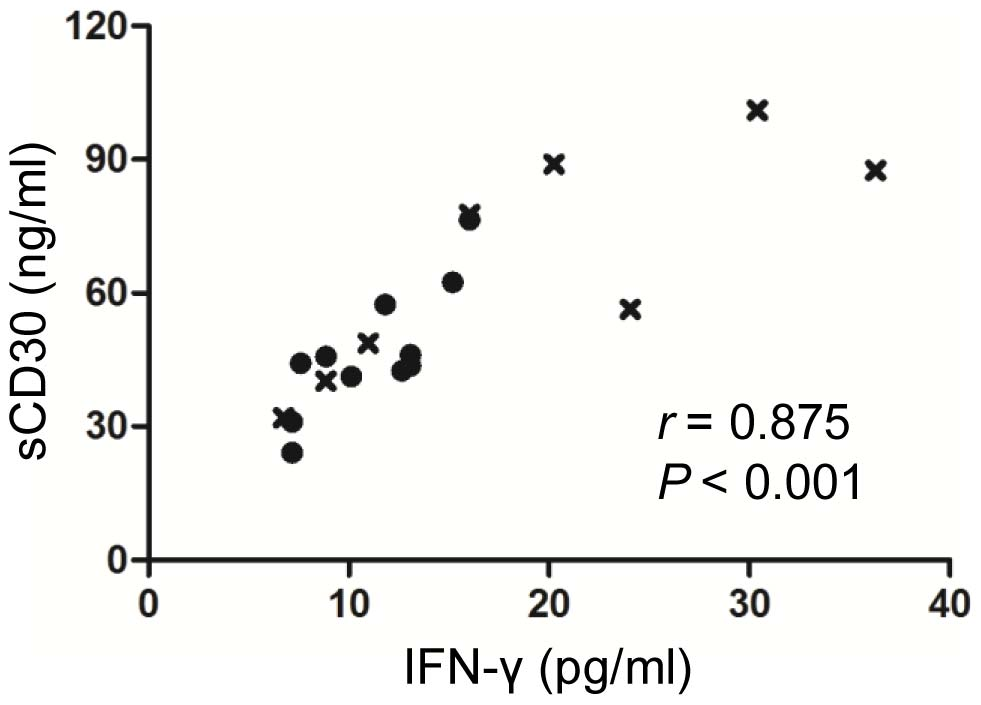
Correlation of BM IFN-γ levels with BM sCD30 levels in SAA patients (•, n = 11) and VSAA patients (×, n = 8).

CD30 was identified as a marker of IFN-γ-producing T cells, so we further determined BM plasma IFN-γ levels in patients with SAA (including VSAA), and found a positive correlation between sCD30 levels and IFN-γ levels (Figure 3), which indicated enhanced CD30 signaling might lead to increased production of IFN-γ in patients with SAA.

### Cell surface expression of CD30 by SAA CD3^+^ T cells during allogeneic stimulation

**Figure 4:**
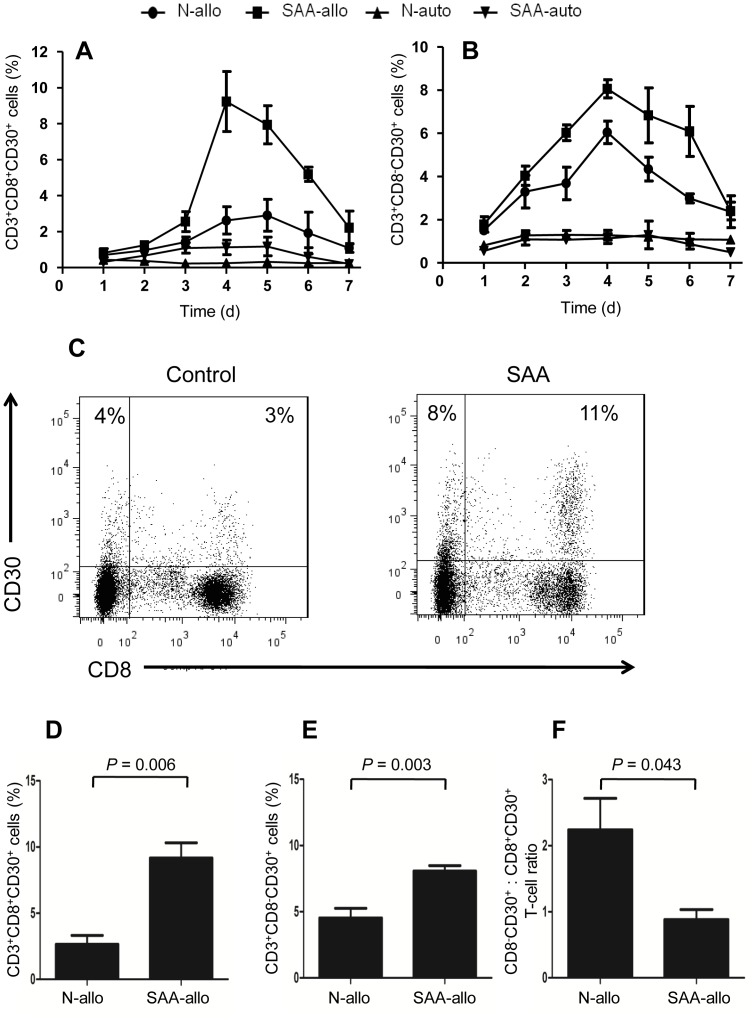
Allogeneic stimulation-induced surface expression of CD30 on T cells. BM CD3^+^ T cells from healthy individuals and SAA patients were co-cultured with mitomycin C treated autologous or allogenetic mononuclear cells for a total of 7 days, kinetics of cell surface expression of CD30 by CD3^+^CD8^+^ T cells (A) and CD3^+^CD8^−^ T cells (B) were determined by FCM. Data represent mean ± SE of three independent experiments. (C) Representative FCM analyses of cell surface expression of CD30 by T cells from healthy individuals and SAA patients after allogeneic stimulation. Results at day 4 when the maximum CD30 expression reached were shown. (D) and (E) Cells surface expression of CD30 by allogeneic stimulated T-cell subsets at day 4 were determined in 5 healthy individuals and 6 SAA patients. (F) CD3^+^CD8^−^CD30^+^: CD3^+^CD8^+^CD30^+^ T-cell ratios in healthy individuals and in SAA patients

We had found elevated BM sCD30 levels in SAA, so next we wanted to explore whether T cells from SAA patients expressed more CD30 when exposed to alloantigen compared to those from healthy individuals. To answer this issue, we determined the induction kinetics of CD30 expression on T-cell subsets from SAA patients and healthy individuals after autologous and allogeneic stimulations. The significantly increased percentages of CD30-expressing CD3^+^CD8^+^ (Figure 4A) and CD3^+^CD8^−^ (Figure 4B) T cells in SAA patients as well as in healthy individuals after allogeneic stimulation instead of autologous stimulation were observed. In both SAA patients and healthy individuals, cell surface expression of CD30 by T cells reached peak at day 4 or day 5 during allogeneic stimulation, then quickly decreased to a very low level. Importantly, the significantly higher percentages of CD30-expressing CD3^+^CD8^+^ and CD3^+^CD8^−^ T cells in SAA patients than those in healthy individuals, especially at day 4 were also observed (Figures 4C-E). Furthermore, we found that CD3^+^CD8^−^ T cells expressed more CD30 than CD3^+^CD8^+^ T cells in healthy individuals, whereas the expression levels of CD30 were comparable between CD3^+^CD8^−^ T cells and CD3^+^CD8^+^ T cells in patients with SAA, as shown by the significantly higher CD3^+^CD8^−^CD30^+^: CD3^+^CD8^+^CD30^+^ T-cell ratios in healthy individuals in comparison with patients with SAA (Figure 4F).

### Release of sCD30 and IFN-γ by SAA CD3^+^ T cells during allogeneic stimulation

**Figure 5:**
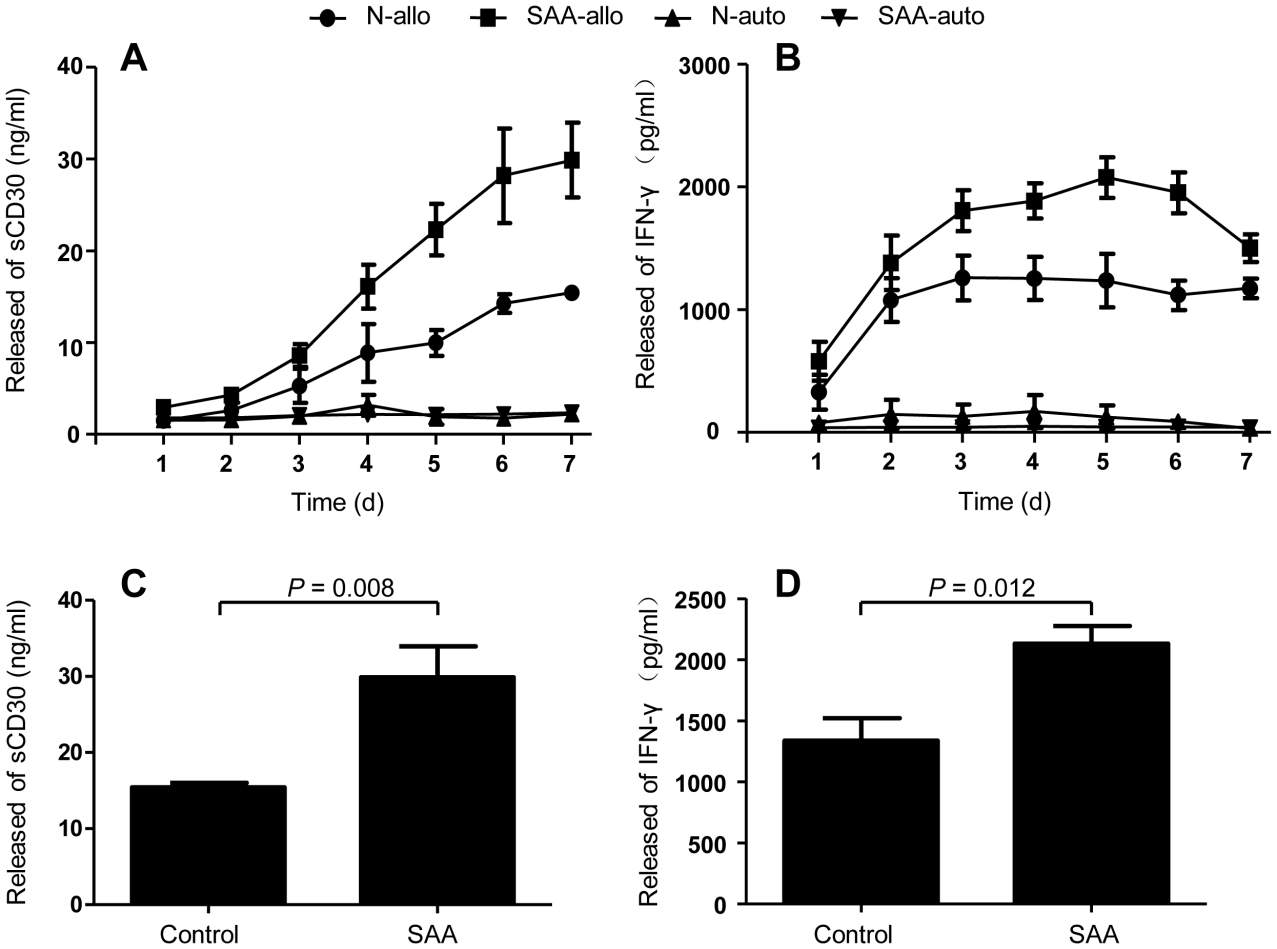
Allogeneic stimulation-induced release of sCD30 and IFN-γ by T cells. BM CD3^+^ T cells from healthy individuals and SAA patients were co-cultured with mitomycin C treated autologous or allogenetic mononuclear cells for a total of 7 days, kinetics of sCD30 (A) and IFN-γ (B) released into culture supernatants were analysed by ELISA. Data represent mean ± SE of three independent experiments. (C) and (D) Released sCD30 and IFN-γ by allogeneic stimulated T cells at day 4 were determined in 5 healthy individuals and 6 SAA patients.

Surface CD30 can be cleaved as soluble form, so we measured the kinetics of sCD30 and IFN-γ released into the culture supernatants by autologous and allogeneic stimulated CD3^+^ T cells. In line with CD30 expression on T cells, significantly increased levels of sCD30 were detected in culture supernatants from allogeneic stimulated T cells but not from autologous stimulated T cells (Figure 5A). After allogeneic stimulation, sCD30 levels were significant higher in patients with SAA than in healthy individuals (Figure 5C). Distinct correlation was observed between maximum sCD30 level (reached at day 7) and CD30 expression on T cells at day 4 instead of at day 7 (Table 1), when most CD30 had been cleaved from T cells surface.

**Table 1:** Maximum sCD30 level or IFN-γ level positively correlates with the percentage of CD30^+^ T cells at day 4 but not day 7 after allogeneic stimulation.

	CD30^+^ T (%)			
	d4		d7	
	r	p	r	p
sCD30	0.886	0.019	0.23	0.661
IFN-γ 0.018	0.889	0.018	0.126	0.812

Spearman's rank correlation test.

Significantly elevated levels of IFN-γ were detected in allogeneic cultures but not in autologous cultures (Figure 5B). After allogeneic stimulation, IFN-γ levels were significantly higher in SAA patients than those in healthy individuals (Figure 5D). Once again, specific correlation between maximum IFN-γ levels (reached at day 4 or day 5) and CD30 expression on T cells was observed at day 4 instead of day 7 (Table 1).

### Production of IFN-γ by allogeneic sitmulated CD30^+^ T cells

**Figure 6:**
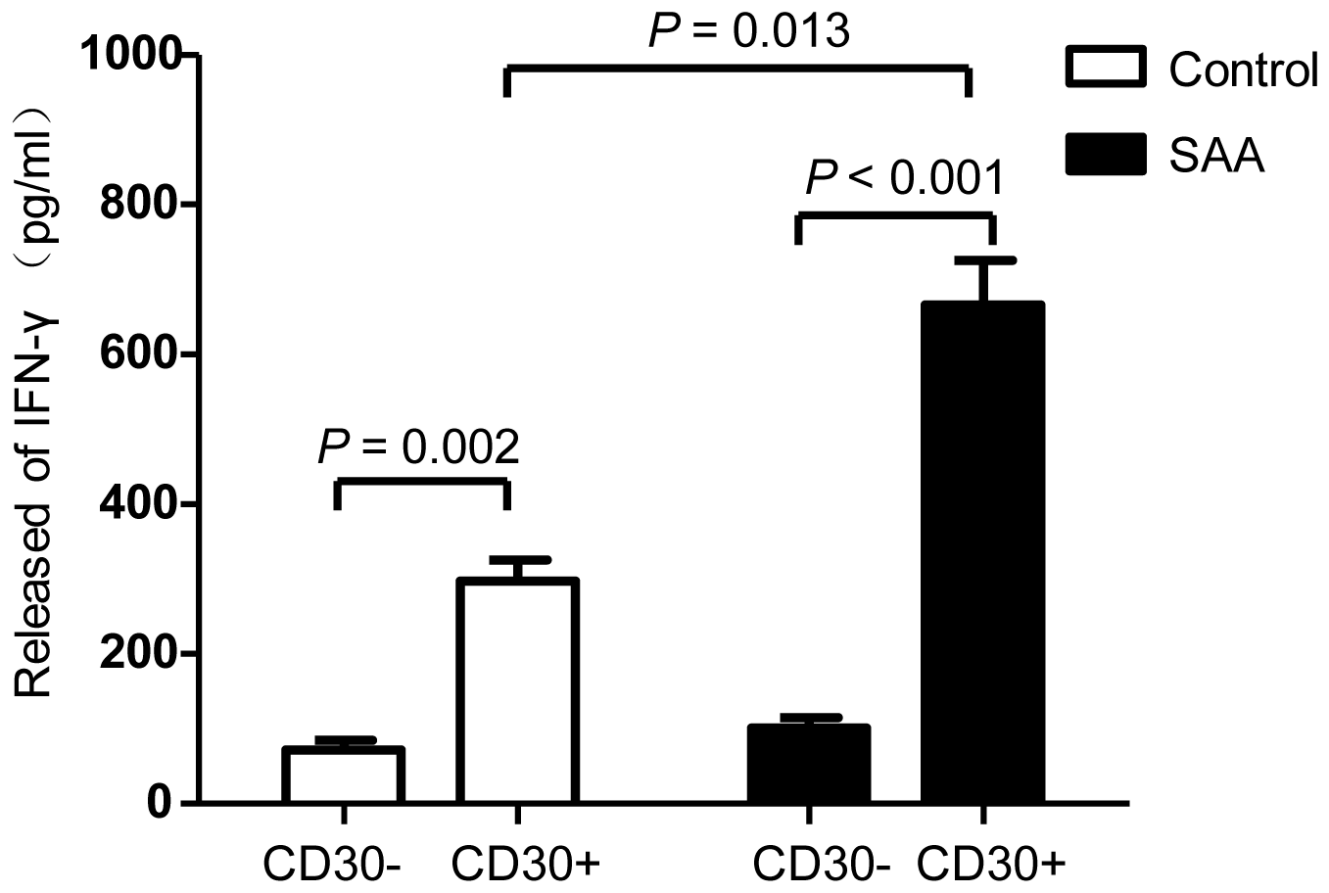
Relationship between CD30 and IFN-γ. After 4 days of culture, allogeneic stimulated T cells were sorted by FACS, CD30^+^ and CD30^−^ T cells were separately cultured for additional 2 days, then IFN-γ levels in the culture supernatants were determined by ELISA. Data represent mean ± SE of three independent experiments.

To assess IFN-γ-producing capacity of induced CD30^+^ T cells from SAA patients, allogeneic stimulated T cells were separated by FACS day 4 after culture. CD30^+^ and CD30^−^ T cells were separately cultured for additional 2 days, followed by determining the levels of IFN-γ in the culture supernatants by ELISA. CD30^+^ T cells produced significant more IFN-γ than did CD30^−^ cells in SAA patients as well as in healthy individuals (Figure 6). Importantly, induced CD30^+^ T cells from SAA patients produced more IFN-γ than healthy individuals.

### Association between IST and sCD30

**Figure 7:**
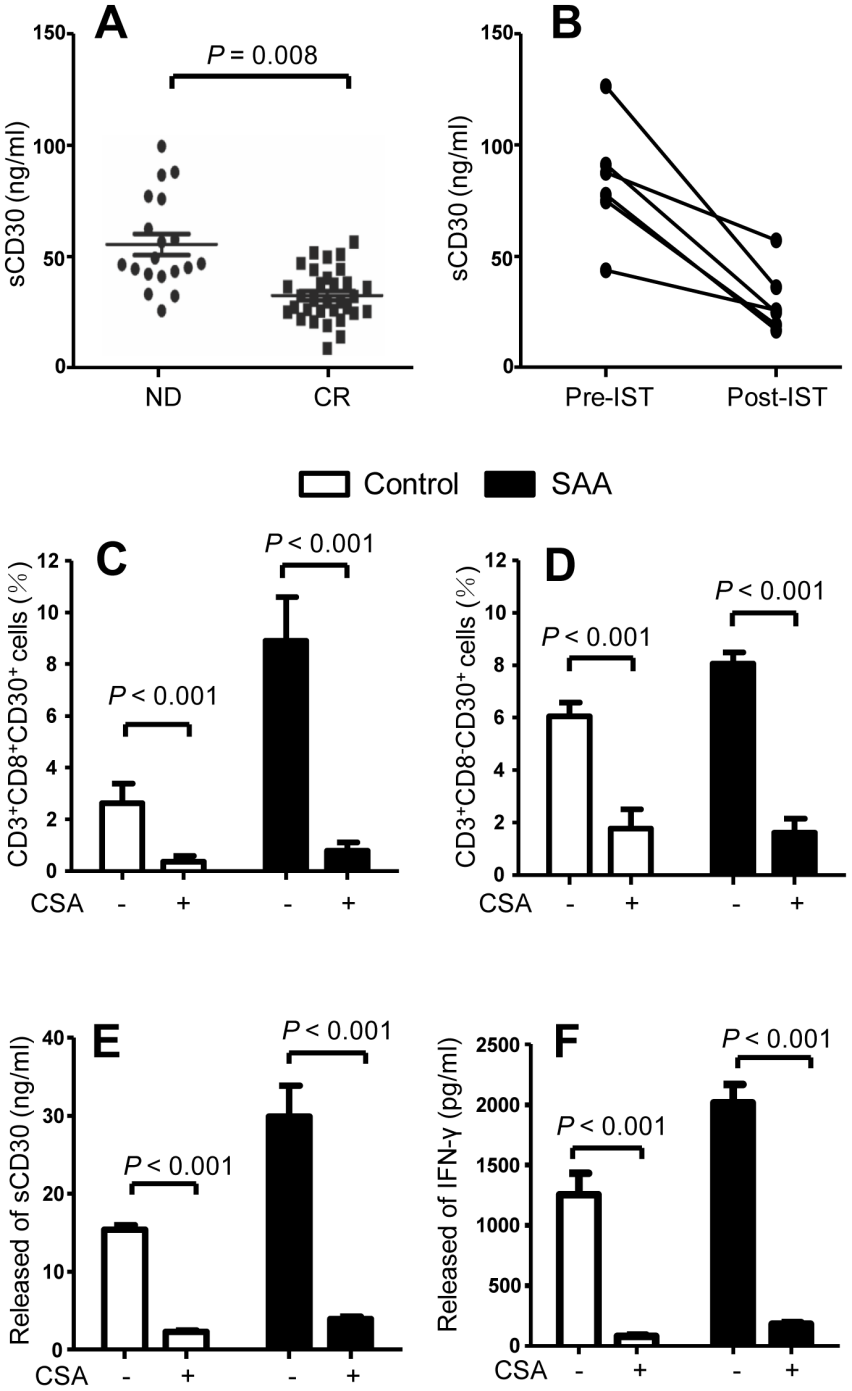
Relationship between BM plasma sCD30 levels and IST. (A) BM plasma sCD30 levels in SAA patients at diagnosis (n = 19) and in CR (n = 24). (B) BM samples pre- and post-IST were collected from 6 SAA patients, BM plasma sCD30 levels were determined by ELISA. CsA was added at 200 ng/mL to the allogeneic stimulated cultures, cells surface CD30 expressions (C and D) and released sCD30 (E) and IFN-γ (F) into culture supernatants were analysed at day 4 by FCM and ELISA, respectively. Data represent mean ± SE of three independent experiments.

In order to investigate the association between sCD30 and disease activity, we detected the sCD30 levels in patients with SAA (including VSAA) at diagnosis (n = 29) and in CR (n = 24). Patients in CR had significantly lower levels of sCD30 than patients at diagnosis (Figure 7A). BM samples pre- and post-IST were collected from 6 SAA patients who had responded to IST, all patients had significant decrease in sCD30 levels after successful IST (Figure 7B).

To test the direct effect of CsA on CD30 expression, we added CsA at 200 ng/mL to the allogeneic cultures. Surface CD30 expression on T cells, and levels of sCD30 and IFN-γ in culture supernatants were analysed at day 4. CsA significantly inhibited CD30 expression on CD3^+^CD8^−^ (Figure 7C) and CD3^+^CD8^+^ T cells (Figure 7D) of both SAA patients and healthy individuals. CsA also reduced sCD30 (Figure 7E) and IFN-γ (Figure 7F) in the culture supernatants from T cells of both SAA patients and healthy individuals.

## Discussion

Elevated amounts of sCD30 in blood have been reported in many autoimmune diseases. This is the first study to investigate the role of CD30 in the pathogenesis of AA. We found increased BM plasma levels of sCD30 correlated well with disease severity, CD4/CD8 T-cell ratio and BM plasma levels of IFN-γ in SAA. More interestingly, higher plasma level of sCD30 in BM than in PB was observed in each SAA patient, but not in healthy individual. We also revealed an elevated CD30 expression at mRNA levels in BMMCs of patients with SAA compared with healthy individuals. These data suggested that CD30-associated T cell activation did exist, especially in BM as the target organ of immune attack in patients with SAA. In addition, T cells and in particular CD8^+^ T cells from patients with SAA showed enhanced allogeneic response by the fact that immune cells from these patients could be induced by alloantigen to express more CD30 than that from healthy controls.

Though the physiological function of CD30 remains unclear, it is unambiguous that CD30 is not a general marker of activated T cells but plays a critical role in alloimmune response. CD30 was suggested to participate in eliminating autoreactive T cells in thymus and inducing T cell apoptosis in periphery *in vivo* [31]–[34]. Compared with anti-CD3 plus anti-CD28 antibodies and autologous antigen presenting cell (APC), allogeneic APC induced a greater proportion of CD30^+^ cells which not only were the predominant proliferating T cells in response to alloantigen, but also represented a subset of T cells that were the primary source of IFN-γ *in vitro* [17]. These findings also agreed with the observation that BM plasma levels of sCD30 positively correlated with the levels of IFN-γ in patients with SAA.

Our *in vitro* stimulation experiments unveiled that the proportion of CD30^+^ cells increased and peaked at day 4, then decreased quickly, but the sCD30 levels increased in a time-dependent manner paralleled with high production of IFN-γ. Significant correlation of maximum sCD30 level or maximum IFN-γ level with CD30 expression on T cells was observed at day 4, but no at day 7 when most surface CD30 had been cleaved and released as soluble CD30. This observation, consistent with previous studies [35], implied that surface CD30 was transiently expressed by activated T cells, but sCD30 could exist for a relatively longer period, and was easily quantified to reflect the maximum cell surface CD30 expression. It is intriguing to speculate that potent proliferating of CD30^+^ T cells caused by autoantigens exist abundantly in the early phase of SAA, when the disease still hardly been aware because of lacking obvious clinical symptoms. These CD30^+^ T cells may have a powerful ability to inhibit hematopoiesis because of enhanced production of IFN-γ. During the aggressive stage of SAA, drastic immune attack eliminates most hematopoietic cells baring autoantigens, leads to few new born CD30^+^ T cells which only have a transient existence, but sCD30 cleaved from activated CD30^+^ T cells can circulate in the bloodstream for a relative long time and reflect the degree of T cell activation. This may explain why we can't detect abundant expression of CD30 on T cell surface but elevated sCD30 levels in SAA.

Another interesting observation was the inverse correlation between plasma sCD30 levels and ALC. As we know most SAA patients show activated immune responses and decreased T cell counts. In addition, the baseline ALC is predictive of response at 6 months following IST [2]. The inverse correlation between sCD30 levels and ALC implied that CD30 participated in regulating apoptosis of T cells in SAA patients. However, the role of CD30 in human peripheral T cell apoptosis is still unclear. Only a few studies reported that CD30 regulated apoptosis in human blood eosinophils, anaplastic large cell lymphoma cells and murine CD8^+^ T cells [34], [36], [37]. So If CD30 signaling affects the apoptosis of human T cells needs further study, especially in patients with SAA.

Disturbed T-cell subset balance with low CD4/CD8 T-cell ratio is present in many AA patients. We found the inverted CD4/CD8 T-cell ratio correlated with sCD30 levels in patients with SAA. This association was also reported in patients with common variable immunodeficiency [38]. We tried to explain this observation and found that while CD30 was predominantly expressed on CD3^+^CD8^−^ T cells in healthy individuals after allogeneic stimulation, the expression level of CD30 was comparable between CD3^+^8^−^ T cells and CD3^+^CD8^+^ T cells in AA. CD8^+^ T cells from AA patients expressed more CD30 than that from healthy individuals. Although very preliminary, these findings suggested that activated CD8^+^ T cells with increased proportion and enhanced allogeneic response might partly contribute to the elevated sCD30 in AA. In addition, we found that the release of IFN-γ reached plateau sooner than the release of sCD30 in allogeneic cultures. This could be explained by a very recent report that IFN-γ was able to trigger the release of sCD30 from allogeneic stimulated T lymphocytes [35]. So, while the CD30 pathway regulate the production of IFN-γ by T cells, IFN-γ may also function as a negative feedback modulator of T cell function by promoting CD30 release from T cells.

Correlations of sCD30 levels with disease activity/severity were reported in patients with systemic lupus erythematosus [22]. Increased levels of sCD30 in serum and synovial fluid were also observed in patients with rheumatoid arthritis [20]. So, sCD30 may be an indicator of immune activation in autoimmune diseases, including AA. Recently, CD30 has been investigated in acute GvHD after HCT [23]–[24]. The well-known pathophysiology of acute GvHD is clear that donor-derived T cells driven by antigen-present cell participated in the genesis of aGVHD, and minor histocomatibility antigens play a critical role [39]. As a marker of alloimmune responses, the elevated amounts of sCD30 in blood decreased after successful IST in SAA, which was similar with aGvHD. Brentuximab vedotin, an antibody-drug conjugates targeting CD30, has been approved by FDA for treatment of relapsed/refractory Hodgkin lymphoma and anaplastic large cell lymphoma [27]–[28]. Besides in hematopoietic cancers, Brentuximab vedotin is now being tested in prevention of GvHD after mismatched unrelated allogeneic HCT and treatment of refractory chronic GvHD. So, considering the increased expression of CD30 in many autoimmune diseases, and the treatment effect of CD30 antibody in type-I sensitized mice [40], this drug may have an application prospect in these diseases with elevated levels of sCD30.

## Conclusion

In summary, we found that sCD30 plasma levels were increased in AA and correlated with disease severity, IFN-γ levels and CD4/CD8 T-cell ratio. T cells and in particular CD8^+^ T cells which had increased proportion and enhanced allogeneic response partly contributed to the elevated levels of sCD30 and IFN-γ in AA. CD30 as a molecular marker that transiently express on IFN-γ-producing T cells, may participate in mediating bone marrow failure in AA. Our results shed more insight into the immune pathology involved in SAA. And in light of the importance of IFN-γ in the pathogenesis of SAA, CD30 may be a potent therapeutic target for SAA. CD30-directed drugs, for example, Brentuximab vedotin, should be tested *in vitro* and in AA mouse model for its ability to suppress activation of T cells by alloantigen.

## Supporting Information

Figure S1 **Expression of CD30 on T cells after allogeneic stimulation.** Representative FCM analyses showed the kinetics of cell surface expression of CD30 by CD3^+^CD8^+^ T cells and CD3^+^CD8^−^ T cells after allogeneic stimulation. BM CD3^+^ T cells from healthy individuals and SAA patients were co-cultured with mitomycin C treated allogenetic mononuclear cells for a total of 7 days.
(TIF)

Figure S2 **CD30 positive T cells were mainly contained in the cell population with larger FSC and SSC.** Representative FCM analyses showed the cell surface expression of CD30 on T cells at day 4 after allogeneic stimulation.
(TIF)

Figure S3 **CsA inhibited the expression of CD30 on T cells after allogeneic stimulation.** Representative FCM analyses showed the cell surface expression of CD30 on T cells at day 4 after allogeneic stimulation.
(TIF)

Figure S4 **Correlation of BM plasma sCD30 levels with baseline RET in AA patients (n = 19).**
(TIF)

Table S1 **Characteristics of patients.**
(DOCX)
