# Supplementary figures and images for: Increased Bone Marrow (BM) Plasma Level of Soluble CD30 and Correlations with BM Plasma Level of Interferon (IFN)-γ, CD4/CD8 T-Cell Ratio and Disease Severity in Aplastic Anemia
Source: PLoS One. 2014 Nov 10;9(11):e110787. doi: 10.1371/journal.pone.0110787 (PMC4226501; doi:10.1371/journal.pone.0110787)

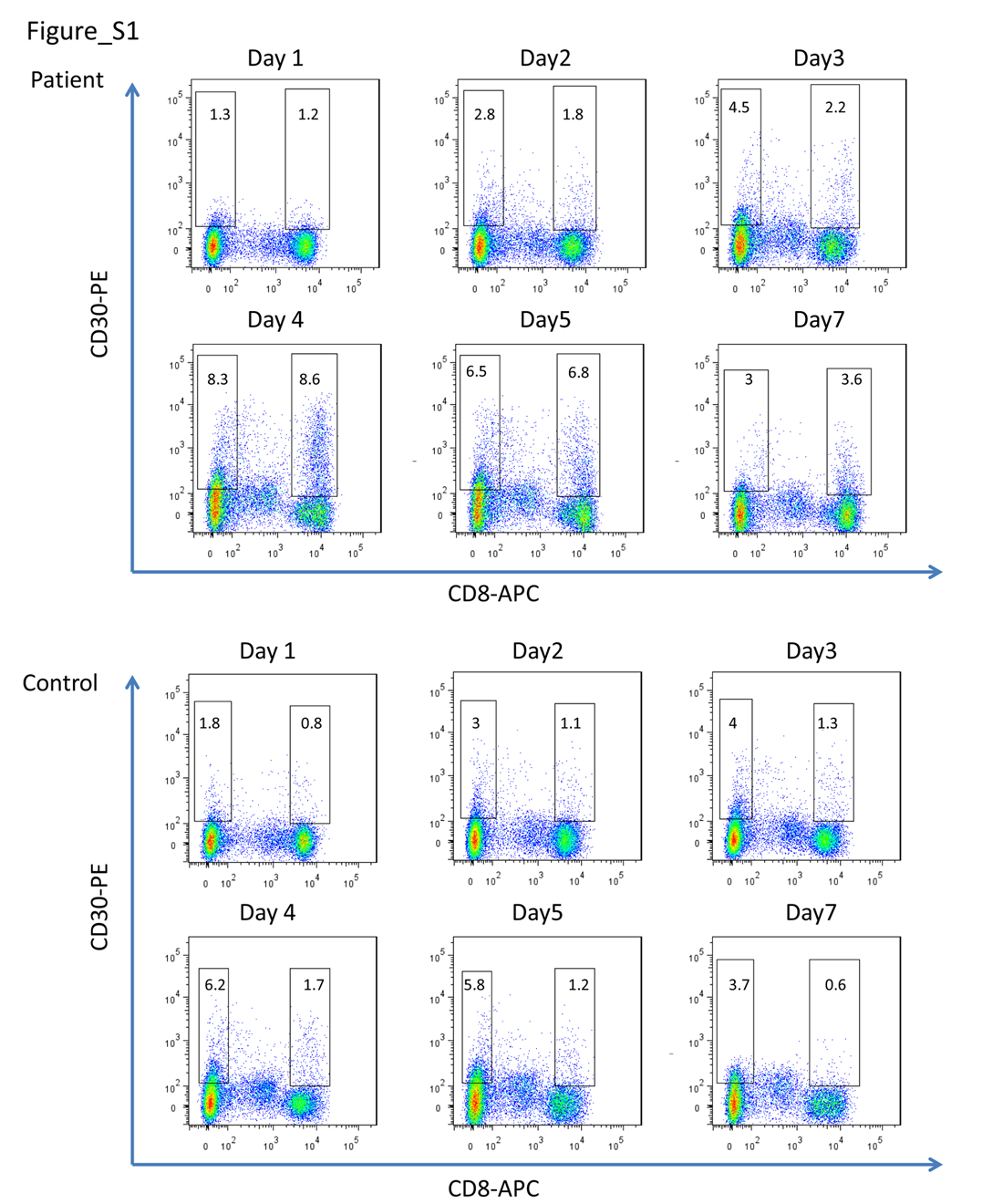

Supplement: Figure S1 — Expression of CD30 on T cells after allogeneic stimulation. Representative FCM analyses showed the kinetics of cell surface expression of CD30 by CD3+CD8+ T cells and CD3+CD8− T cells after allogeneic stimulation. BM CD3+ T cells from healthy individuals and SAA patients were co-cultured with mitomycin C treated allogenetic mononuclear cells for a total of 7 days. (TIF) [file pone.0110787.s001.tif]

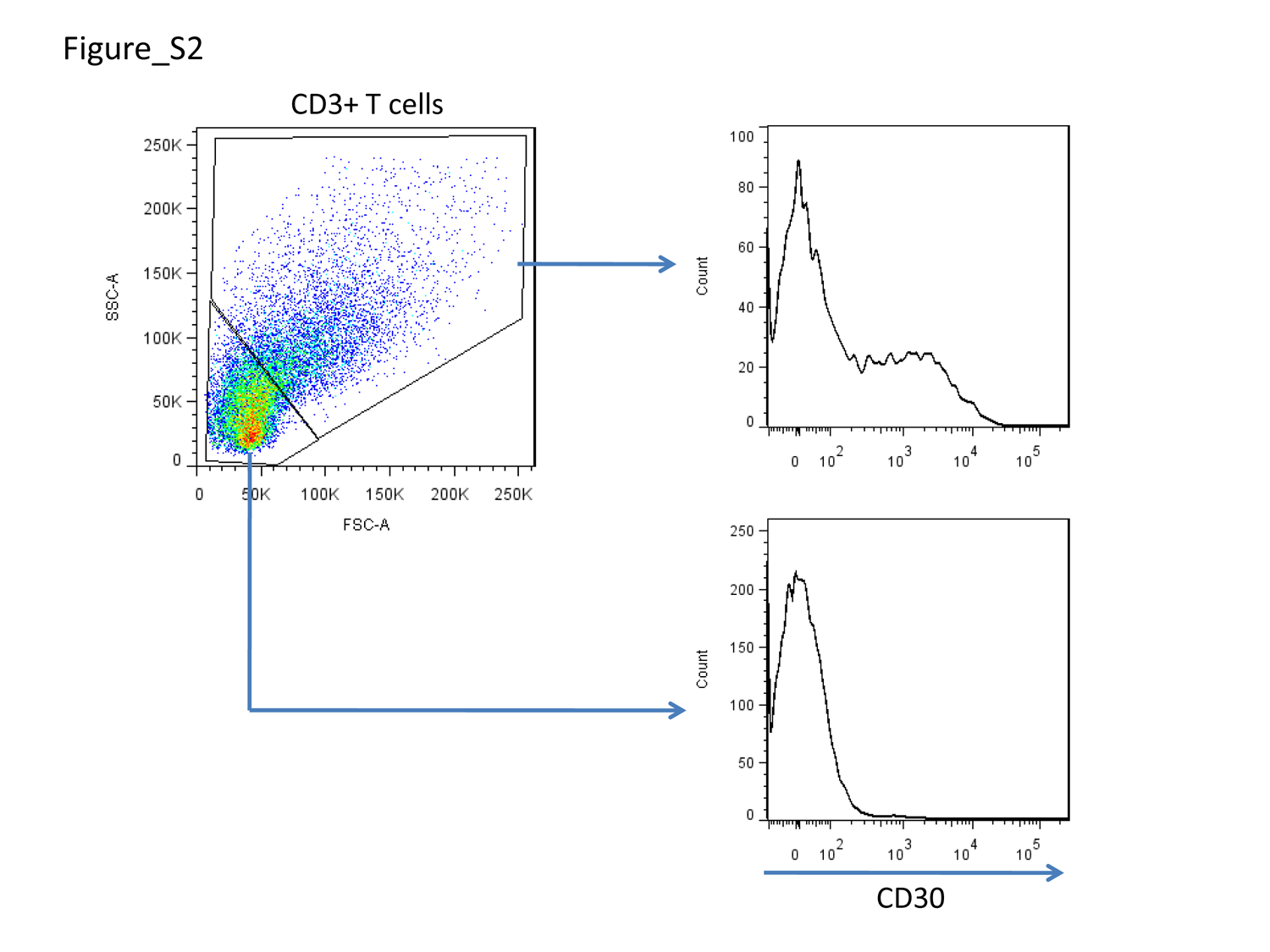

Supplement: Figure S2 — CD30 positive T cells were mainly contained in the cell population with larger FSC and SSC. Representative FCM analyses showed the cell surface expression of CD30 on T cells at day 4 after allogeneic stimulation. (TIF) [file pone.0110787.s002.tif]

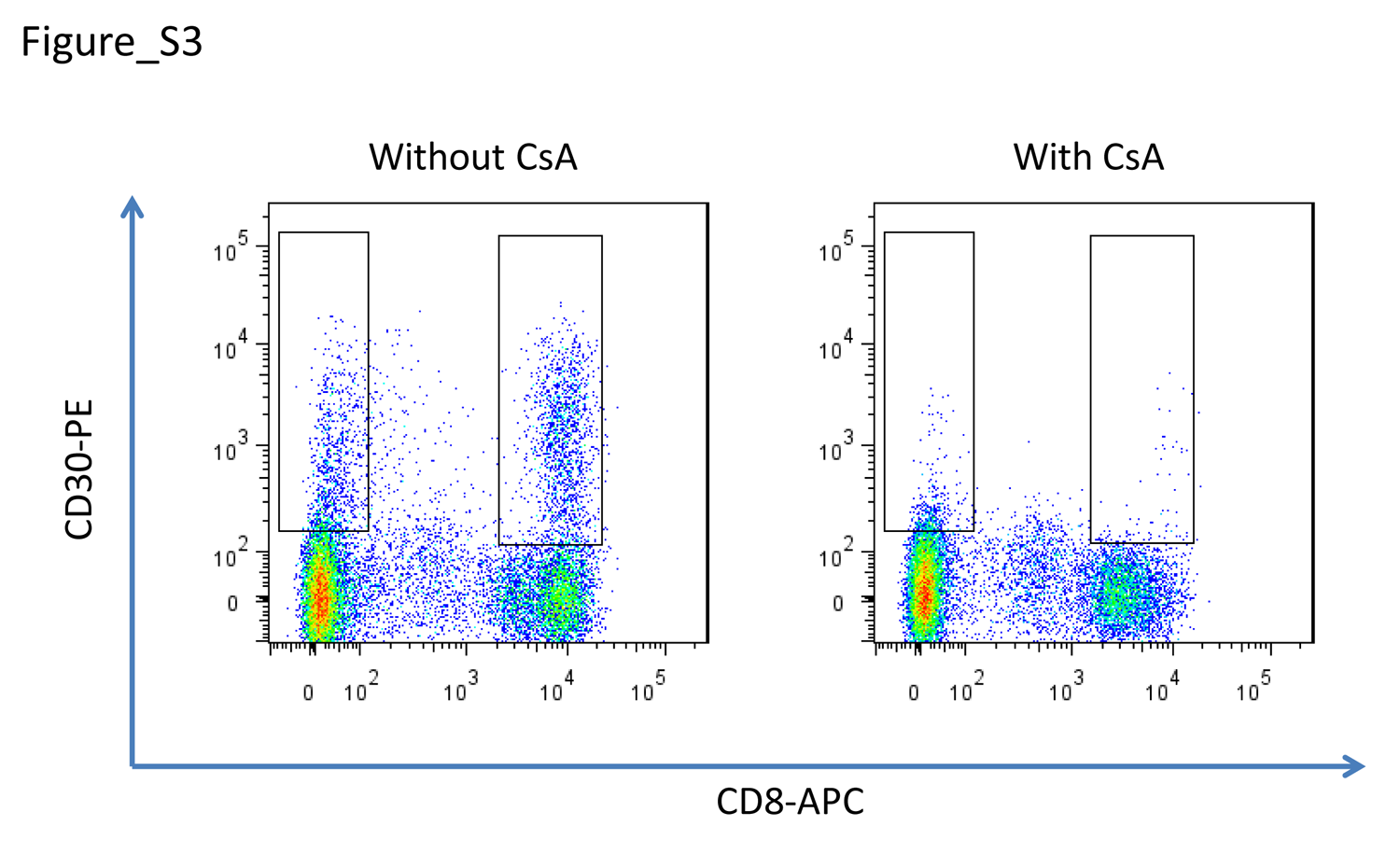

Supplement: Figure S3 — CsA inhibited the expression of CD30 on T cells after allogeneic stimulation. Representative FCM analyses showed the cell surface expression of CD30 on T cells at day 4 after allogeneic stimulation. (TIF) [file pone.0110787.s003.tif]

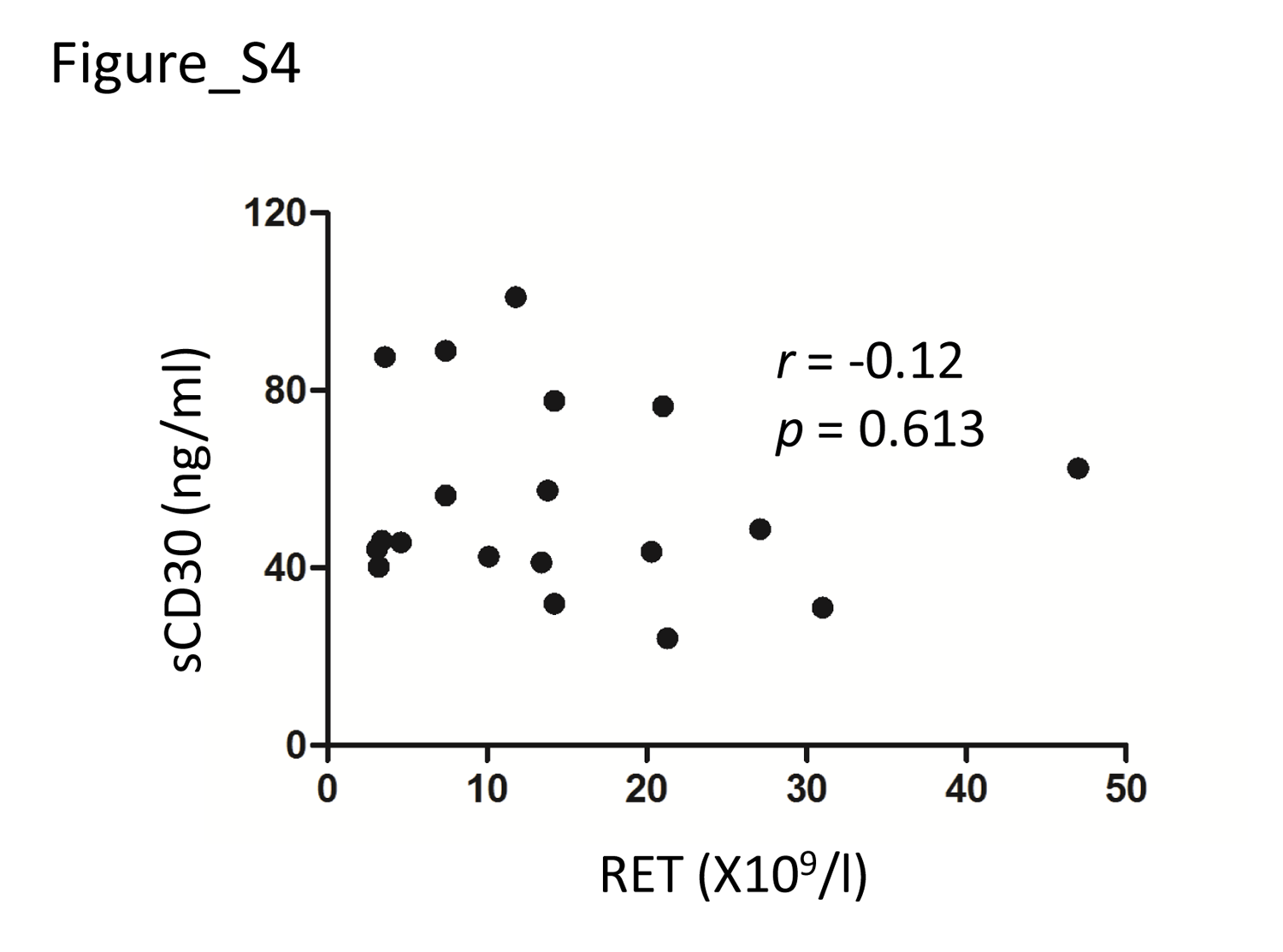

Supplement: Figure S4 — Correlation of BM plasma sCD30 levels with baseline RET in AA patients (n = 19). (TIF) [file pone.0110787.s004.tif]
