# Supplementary material for: Increased Bone Marrow (BM) Plasma Level of Soluble CD30 and Correlations with BM Plasma Level of Interferon (IFN)-γ, CD4/CD8 T-Cell Ratio and Disease Severity in Aplastic Anemia
Source: PLoS One. 2014 Nov 10;9(11):e110787. doi: 10.1371/journal.pone.0110787 (PMC4226501; doi:10.1371/journal.pone.0110787)
Supplement: Table S1 — Characteristics of patients. (DOCX) [file pone.0110787.s005.docx]

| **Table S1. Characteristics of patients** | |
| --- | --- |
| Characteristics | Value |
| Number of patients | 56 |
| Median age, y (range) | 27 (7 - 57) |
| Disease severity at diagnosis (VSAA:SAA:NSAA) | 13:30:13 |
| Sex (M:F) | 29:27 |
| **Treatment and response** |  |
| Newly diagnosed | 32 |
| CR after ATG + CsA | 24 |
| VSAA indicates very severe aplastic anemia; SAA, severe aplastic anemia; NSAA, nonsevere aplastic anemia; M, male; F, female; ATG, anti-thymocyte globulin; CsA, cyclosporin A; and CR, complete response. | |
|  |  |
|  |  |
